# The impact of E-education and innovation on unemployment reduction among graduates: A way forward for higher educational institutes

**DOI:** 10.3389/fpsyg.2022.914104

**Published:** 2022-12-13

**Authors:** Lu Mian, Ridzuan Hussin, Mgr. Gabriela Slaninová, Yusra Shahzadi

**Affiliations:** ^1^Faculty of Education, Languages and Psychology, SEGi University, Petaling Jaya, Selangor, Malaysia; ^2^Faculty of Education, Languages and Psychology, University of Hradec Králové, Hradec Králové, Czechia; ^3^Faculty of Informatics and Management, Department of Management, University of Hradec Králové, Hradec Králové, Czechia; ^4^Department of Management Sciences, COMSATS University Islamabad, Sahiwal, Pakistan

**Keywords:** E-education, youth, innovation, unemployment reduction, HEI’s

## Abstract

Recently, the use of digital skills as a tool to alleviate unemployment concerns of university graduates has gained considerable attention among governments in developing economies. The present study examines the impact of the E-education program (a digital initiative to train university graduates to get self-employed) on the unemployment reduction of young graduates of higher educational institutes (HEIs). We also explore the mediating influence of innovation in the association between E-education and unemployment reduction. The target population of the study was students who have successfully completed the E-education program. The convenient sampling technique was used for data collection from 416 respondents through a structured questionnaire. Collected data were analyzed using different statistical techniques, such as descriptive statistics (reliability analysis, normality analysis, and correlation) and structural equation modeling for measurement of the structural model. The study findings assert that the E-education program has significant effects on the unemployment reduction of graduates. Furthermore, innovation also significantly mediates the association between E-education and reduce unemployment among students. It implies that the HEIs should also roll out E-education programs, which would eventually help reduce unemployment and promote the growth of the E-education industry in the country. Finally, policy prescriptions are discussed on the role of an E-education-driven innovation to curtail unemployment in developing countries.

## Introduction

Cross-border e-commerce trade activities have witnessed a remarkable surge around the globe in recent years (Cheng et al., 2019). The internet has now penetrated into the economic and social domains of societies (Guo et al., 2019). The rapid digital advancement of e-commerce has necessitated universities to introduce a collaborative education system that incorporates e-commerce and online skills training to maximize the employment prospects of students (Wang and Tang, 2020). Considering the rapid growth of online businesses, universities are now keen to introduce e-commerce and digital skill-based training as study majors to gain a competitive advantage in this niche. Besides, the use of virtual laboratories to simulate actual job experience is gaining traction in the education industry (Vejačka, 2019).

**FIGURE 1:**
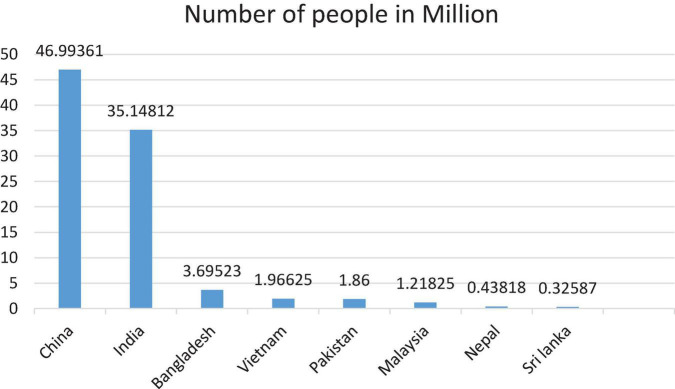
Number of people enrolled in tertiary education in selected Asia-Pacific economies, 2019.

Against the backdrop of sluggish economic growth, youth unemployment is one of the critical concerns for governments. While a disproportionate share of youth in unemployment and a considerably higher ratio of young people in emerging countries provide an opportunity as well as pose a challenge to the country’s future. The opportunity emerges from the fact that youth can serve as an engine of development as they have the energy and will to transform a country’s future (UNDP, 2018). Contrarily, failure in the provision of job opportunities to young people as they enter the working age-group may lead to a demographic disaster (Mitra and Verick, 2013). Figure 1 shows the state of tertiary education in the Asia-Pacific region. China is leading the industry with a clear margin, while in Pakistan, only 1.86 million people are enrolled in tertiary education.

**FIGURE 2:**
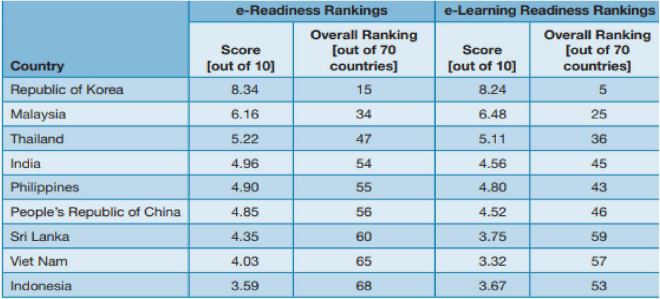
Asian E-readiness rankings and E-learning readiness rankings. Source: Adapted from EIU (2003, 2008) as reported in Latchem and Jung (2009).

In the past two decades, information technology (IT) has assumed a central role in the emergence of the knowledge-based economy and has emerged as a key indicator of economic growth for many leading economies of the world. It also creates highly skilled jobs (Avom et al., 2021), while changes in youth unemployment are positively related to domestic IT industry production (Dube et al., 2015). Figure 2 depicts the e-readiness and e-learning rankings of the top nine Asian countries. Pakistan is not a part of the list. Nonetheless, Pakistan’s ranking in the business-to-consumer e-commerce index is 114 out of 152 countries.

Given the massive share of youth in Pakistan and their keen interest in the IT field, since 2012, the country’s entrepreneurship and digital innovation landscape have grown exponentially. Thus, considering the 2-fold challenge of creating new jobs and, at the same time, bridging the IT skills gap among youth, on 19 May 2017, the government launched the E-Rozgar training program (hereinafter, E-education). This program aims to provide internet-based freelancing training opportunities to youth to curtail unemployment and steer the country’s economic growth. E-education is not an entitlement program, but rather a minimum eligibility criterion that must be fulfilled by the potential candidates. The students must have completed 16 years of education and should be in the age group of 22–35 years to be enrolled in this program. The vision of the program is to “reduce unemployment and drive economic growth in the country by boosting inflows of foreign currency.”

The skill development program is an essential step in providing training along with employment opportunities. It provides human capital to society and increases the income levels of individuals. King (2009) postulated that skill development and technical training are becoming increasingly powerful policy tools in developing countries to promote economic growth. Khan et al. (2010) asserted that governments should initiate new programs in collaboration with global donors to develop self-employment skills in youth. These training programs can help impoverished students to obtain jobs, increase their earning levels, and fulfill their basic needs to live a decent life. Besides, the governments should also set up various skill development programs for youth to enhance their skills that help reduce rampant unemployment (Audu et al., 2013).

Keeping this in view, several skills development programs have been initiated by the government. However, despite the fact that the government is spending a large amount of funds on such schemes, systematic evaluations of their effects on unemployment reduction and the personal financial management of trainee students are very limited. This article is an attempt to fill this void by exploring the quantitative data, which are collected through a structured questionnaire from the students who have successfully completed the government’s E-education program.

Apart from this, it is observed that fundamental skills development and vocational knowledge are imperative for innovation as they promote new abilities to learn, change, and be creative at work (Dalitz et al., 2011). Incessant training ensures access to cutting-edge knowledge and augments the propensity to innovate in a society (Bauernschuster et al., 2009). Similarly, both classroom training and on-the-job training are required to boost the innovation capacity of the students (Dostie, 2018). This evidence sheds light on the value of training programs in enhancing the scale of innovation that has ultimate positive implications on the expansion of employment opportunities for the youth. Therefore, the present study also examines the mediating role of “innovation” between the E-education program and unemployment reduction in youth. The research contributed to the E-education literature by providing empirical evidence of the effects of this type of program in a new dimension (innovation).

Notwithstanding the fact that current research focuses on one program (i.e., E-education) offered by the government of Punjab, Pakistan. It has wider implications for similar programs offered in various parts of the country by educational institutions and the government to curtail unemployment in the youth.

The rest of the article is arranged as follows: Section “Literature review and hypotheses development” presents the relevant literature and develops hypotheses, Section “Materials and methods” presents data and methods, Section “Empirical findings” presents the results and discussions, and the final section “Discussion” concludes the study.

## Literature review and hypotheses development

### E-education program and unemployment

Unemployment is a situation when people are skilled and willing to do work but are unable to get a suitable job (Fajana, 2000). The menace of persistent unemployment creates a plethora of psychological ailments among graduates (Peterie et al., 2019). Especially the governments in developing countries are facing a serious issue of graduated unemployed youth (Sever and İğdeli, 2018). In the current era of competition in the higher education arena, educational institutions produce thousands of graduates each year but not all of them are securing suitable jobs. Moreover, one of the key reasons for unemployment among youth is the lack of requisite skills required by the employers in today’s job market (Epure and Barna, 2021). However, if the government and higher educational institutions effectively equipped these unemployed graduates with enterprise abilities, they would have become job creators rather than job seekers (Okoye, 2017).

Moreover, e-business skills have the potential to generate incredible new capital, generally through entrepreneurial start-ups and commercial ventures. It is also transforming the rules of competition for established businesses in an extraordinary way. Therefore, in the past few decades, several scholars have shifted their focus toward e-business, innovation, and creativity (Amit and Zott, 2001; Amuna et al., 2019; Epure and Barna, 2021). Similarly, electronic commerce encompasses sharing business information, maintaining business relationships, and conducting business transactions through internet-based technology (Maqbool et al., 2013; Mgunda, 2019). A positive association between telecommunications and economic growth is well documented in the literature (Gómez-Barroso and Marbán-Flores, 2020).

Keeping in view the importance of e-skills, many independent organizations and governments have launched training programs to equip youth with new skills. These programs provide cutting-edge E-education to people and create employment opportunities and are thus considered one of the major factors to reduce unemployment (Emeh, 2012).

Noreen (2011) observed that the training program has a significant positive impact on the income, education, and employment level of youth. Nevertheless, only highly skilled and trained workers would be able to deal with the changing business environment as they are capable of creating sustainable development for youth (Wallenborn, 2009). Training and skills development programs help considerably change the employment rate over time (Amjad and Kemal, 1997). Besides, internet based skills development programs not only improve the capabilities of the trainees but also generate new employment avenues (Layard et al., 2005). Moreover, ICT penetration in a society lowers the unemployment rate and helps grow the economy of a country (Hussain et al., 2021; Mossberger et al., 2021). Professional learning and training contribute to accomplishing national development goals as skillful individuals would be more creative in boosting their employment and income (Jones, 2007).

In a similar vein, Fanati and Manfredi (2003) revealed a negative association between skills development and the unemployment rate. They argued that when people get training or skills from any program or educational institution, their knowledge and skills are optimized, which ultimately enables them to secure a good job promptly. Training programs (like E-education) help reduce unemployment among youth (Joshua et al., 2015). All these lines of evidence support the notion that training and skills development programs such as E-education program help reduce the unemployment rate among students. Thus, we hypothesize that;

H1: E-education program has a positive association with unemployment reduction among students.

### E-education program, innovation, and unemployment

Innovation is a process of transforming thoughts into opportunities and putting these opportunities into reality. Innovation is concerned with improving how institutions deliver products or services (Baskaran and Mehta, 2016). Moreover, the term innovation consists of two diverse stages: The first stage is a creative process that grasps the generation of innovative ideas. Whereas, the second stage is implementing the idea that describes the realistic behavior to create new sources of income. Most unemployed people enhance their knowledge and creativity through education and training, which help them availing new employment opportunities (Utsch and Rauch, 2000). Innovation also explodes through proper training in online working, which ultimately helps reduce the unemployment rate in the country (Carbonell and Rodriguez, 2006). ICT-related education and skills are crucial enablers for breeding innovation and competitiveness (Hüsing et al., 2013). Both classroom and on-the-job training of graduates can lead to extensive product and process innovation (Dostie, 2018). Similarly, Børing (2017) posits that although a positive association exists between employee training and innovation activities. However, this association tends to be indirect mainly because of other human resource practices that are mainly associated with innovation strategies.

Solow (1957) was considered the pioneer who contended that persistent growth in per capita income is a function of improvement in productivity, which can be attained through technological advancements. This Solow model (also known as the neo-classical growth model) has opened up a new horizon of research on the outcomes of technological innovation both at the firm and the country level. Moreover, economists such as Blanchard (2009) proposed a production function, whereby the level of technological innovation determines the output level for each unit of labor. The model specifies that in the medium term when the actual output surpasses the expected output, the real wages of the labor increase, decreasing the country’s unemployment rate.

Similarly, Harrison et al. (2014) studied the effects of innovation on the employment growth of four European countries: France, Germany, the United Kingdom, and Spain. The results depict that although process innovation dislodges employment, product innovation is positively associated with the employment growth of all the sampled countries. Similar findings were reported by Benavente and Lauterbach (2008), Hall et al. (2008), and Peters (2004). A recent study (Çiftçioğlu and Sokhanvar, 2020) examined the short-run and long-run effects of R&D on the rate of unemployment. The study findings implore that in the long run, R&D tends to lower the unemployment rate; however, in the short run, innovation adversely affects employment creation. Similarly, some earlier studies (Blechinger et al., 1998; Regev, 1998) have also suggested the positive effects of innovation on employment growth.

On the contrary, Brouwer et al. (1993) and Klette and Førre (1998) reported a negative association between innovation and employment opportunities. In the context of developing economies, Maneejuk and Yamaka (2020) found that high innovation brings higher economic growth. Considering the inconsistencies in the relationship between innovation and employment/unemployment, Acemoglu and Restrepo (2018) developed a conceptual framework that proposes that technological innovation could be of two types: The first type is “automation of existing tasks,” which substitutes the labor with the machine and raises the unemployment in the country, and the second type of innovation is “new task creation” in which new skilled labor is required to achieve a competitive advantage and thereby decrease the unemployment.

**FIGURE 3:**
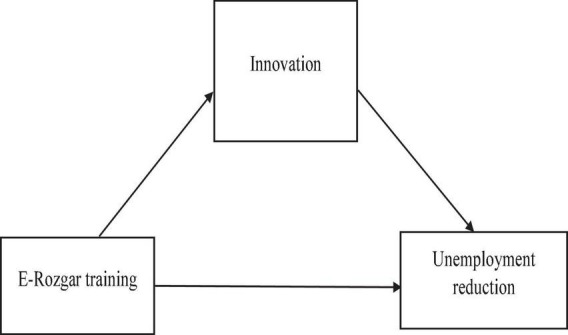
Conceptual framework.

The aforementioned evidence infers a mediating role of innovation between the E-education program and growth in employment opportunities for students (see Figure 3). Therefore, we put forward our second hypothesis:

H2: Innovation mediates the relationship between the E-education program and the unemployment reduction of students.

## Materials and methods

### Sample and data collection

The present study follows a quantitative approach to examine the proposed hypotheses. The primary data were collected by using a survey technique. The population of this study is the students of the E-education program who have successfully completed the program. To obtain an adequate number of responses, we distributed 500 questionnaires using the convenience sampling technique to get maximum responses. A total of 416 students filled the complete survey questionnaires. The response rate was quite high (83%). Demographically, 56% of the respondents (*N* = 233) were men; 53.8% of the respondents (*N* = 224) were 21–25 years old, 31.7% were 26–30 years old, and 14.4% were 31–35 years old. The majority of the respondents received their education in technical (40.6%) and non-technical (34.1%) course tracks, while 25.2% were trained in creative designing.

### Instruments and measures

The instrument of the present research has been adapted from the existing literature with slight modifications to fit the context of the study. The instrument was designed to be completed within 10–12 min to encourage higher participation and completion rates. More precisely, this study employs seven items to measure the quality of the E-education skill development program adapted from Kunche et al. (2011); innovation was measured through four items adapted from Helm et al. (2010) and Burpitt and Bigoness (1997); and the five items that measure unemployment reduction were adapted from Naidoo and Hoque (2017). The response for all 16 items was collected on a 5-point Likert-type scale ranging from 1 = strongly disagree to 5 = strongly agree. We used a 5-point Likert-type scale because it is easier for respondents to complete and thus increases the response rate (Nauman et al., 2019). Moreover, data collected through the 5-point Likert-type scale are more appropriate for confirmatory factor analysis (CFA) and structural equation modeling (SEM) (Deem and Brehony, 2000; Dawes, 2008).

## Empirical findings

### Confirmatory factor analysis

The results of CFA are presented in Tables 1, 2. AMOS 23 was utilized in this study to conduct CFA of the hypothesized model to verify whether the model fit was good and reliable. The results showed that CMIN = 329.78, DF = 116, and CMIN/DF = 2.87, *p* < 0.001 (i.e., CMIN/DF < 3). The model was evaluated with a composite index of overall fitness. The results showed that CFI = 0.94, TLI = 0.93, and RMSEA = 0.06, indicating a good fit.

**TABLE 1:** Measurement model comparison.

	Measurement models	χ^2^	Df	χ^2^/Df	TLI	CFI	GFI	RMSEA
1	E-education program and innovation (2 factor)	1,614.31	123	13.17	0.56	0.59	0.59	0.17
2	Innovation and unemployment reduction (2 factor)	1,790.55	123	14.57	0.50	0.55	0.57	0.18
3	Full model (4 factor)	**329.78**	**116**	**2.87**	**0.93**	**0.94**	**0.91**	**0.06**
	Full model (1 factor)	1,433.06	119	12.08	0.65	0.59	0.61	0.16

Better fit indices are presented in bold; full model (3-factor) combines E-education programs, innovation, and unemployment reduction.

**TABLE 2:** Inter-scale correlation analysis.

S. No		Mean	SD	1	2	3
1	E-education program	1.81	0.56	(0.84)		
2	Innovation	1.99	0.85	0.44**	(0.91)	
3	Unemployment reduction	2.06	0.83	0.44**	0.58**	(0.89)

*n* = 416; alpha reliabilities are presented in parentheses. **p < 0.01.

First, we performed the inter-correlation and reliability test of study variables (refer to Table 2). The result revealed that the E-education program is positively correlated with innovation (*r* = 0.44, *p* < 0.01) and unemployment reduction (*r* = 0.44, *p* < 0.01). Innovation was positively correlated with unemployment reduction (*r* = 0.58, *p* < 0.01). The results of Cronbach’s alpha were also reported, and measurement scales of all the variables were reliable, having a value of above 0.70, as recommended (Nunnally, 1978).

### Structural model

For hypothesis testing, we used the SEM technique in the present study. The model fit indices of the structural model indicate an acceptable model fit as the value of chi-square (χ^2^/df = 2.89), CFI = 0.941, TLI = 0.931, RMSEA 0.067, and SRMR = 0.046 meet the criterion of Hair et al. (2010). Table 3 shows the estimated paths and other statistics of the structural model. The estimated paths are also shown in Figure 2. Hypothesis 1 stated that E-education positively related to unemployment reduction. The results revealed that the E-education program positively related to unemployment reduction (*b* = 0.23, *t* = 4.43, *p* < 0.01) (refer to Table 3). Thus, H1 is supported.

**TABLE 3:** Structural equation model results.

S. No	Variable	*B*	SE	*T*	*P*
1	E-education program → Unemployment reduction	0.23	0.05	4.43	0.000
2	E-education program → Innovation	0.47	0.04	10.32	0.000
3	Innovation → Unemployment reduction	0.52	0.04	11.50	0.000
Indirect effect	E-education program → Innovation → Unemployment reduction	0.24	0.03	7.71	0.000

*n* = 416. Path-1 = IV→DV, Path-2 = IV→MV, Path-3 = MV→DV, Path-4 = IV→MV→DV.

Hypothesis 2 proposes that innovation mediates the relationship between E-education and unemployment reduction. The result showed that E-education has a positive indirect effect on unemployment reduction *via* innovation (coefficient = 0.24, *p* < *0.001*), which supports Hypothesis 2.

The indirect effect was examined using the MPLUS delta method. This method is considered an alternative to the Sobel test and has similar robustness and statistical power to explain the indirect effects (Junaid et al., 2019). Therefore, the findings of the present study do not require any additional validations through the Sobel test.

## Discussion

This study focuses on one of the most prominent programs, “E-education,” which targets educated youth to create employment opportunities in the era of the internet of things. The aim of the present study was to find a link between E-education and unemployment reduction *via* innovation. The existing literature also highlighted the need to identify mediating mechanisms between the E-education program outcome and the unemployment relationship (Rosemary et al., 2020). We believe that with the help of E-education programs, students will be better equipped, and by utilizing these learned skills, innovation will enhance which leads to the reduction in the unemployment. This happens by making E-education individuals feel that they are capable of earning (unemployment reduction). The current study has taken an E-education program in which trainers teach the students about freelancing, project hunting, and product delivery practices that collectively form a bundle. All these practices are directly beneficial for individual unemployment reduction (Rosemary et al., 2020).

We also invigorate the role of innovation in the relationship between E-education and unemployment reduction. Previous studies highlighted the need to examine the mediating mechanisms that explain the relationship between E-education and outcomes (Børing, 2017; Rampa and Agogué, 2021). The present study findings stated that innovation mediates the relationship between E-education and unemployment reduction. The results are aligned with previous studies (Mohd Rosli and Normayuni Mat, 2019; Ylijoki et al., 2019).

Data were collected from the successfully passed out students of the E-education program. The research contributed to the E-education literature by providing empirical evidence of the effects of this type of program in a new dimension (innovation). The results show strong evidence for all hypothesized relationships; participation in E-education programs leads to unemployment reduction. Furthermore, the study also confirms the mediating effect of innovation on the relationship between E-education and unemployment reduction.

The results presented in this study paint a very optimistic picture of E-education program-induced unemployment reduction *via* innovation. Pakistan is a developing country, with over 26% of its population consisting of youngsters, of which millions are unemployed. Therefore, not only the government should continue this program (to curb unemployment) rather HEIs should also come forward and start this type of E-education program to equip the youth with contemporary skills along with traditional tertiary education. Moreover, keeping in view the exponential growth in Pakistan’s e-commerce market in the past few years, the government should also expand the course tracks offered during the program.

The present study also has some limitations. First, due to the utilization of single-source self-reported information, common method bias (CMB) may have exaggerated the correlations. Thus, future studies may use multi-source in order to mitigate CMB. Second, in the present study, we used innovation as an underline mechanism to explain the relationship between E-education and unemployment reduction. Hence, future studies may use different underline mechanisms to uncover the consequences of E-education programs. Future studies in this domain can draw a regional comparison on the penetration of E-education programs and resultant employment and economic growth prospects. Nevertheless, it would be interesting to draw on a comparison between the use of E-education and skills development in developing and advanced countries.

## Data availability statement

The original contributions presented in this study are included in the article/supplementary materials, further inquiries can be directed to the corresponding author.

## Author contributions

YS wrote the original manuscript. All authors contributed to the article and approved the submitted version.
